# A Snapshot on the On-Label and Off-Label Use of the Interleukin-1 Inhibitors in Italy among Rheumatologists and Pediatric Rheumatologists: A Nationwide Multi-Center Retrospective Observational Study

**DOI:** 10.3389/fphar.2016.00380

**Published:** 2016-10-24

**Authors:** Antonio Vitale, Antonella Insalaco, Paolo Sfriso, Giuseppe Lopalco, Giacomo Emmi, Marco Cattalini, Raffaele Manna, Rolando Cimaz, Roberta Priori, Rosaria Talarico, Stefano Gentileschi, Ginevra de Marchi, Micol Frassi, Romina Gallizzi, Alessandra Soriano, Maria Alessio, Daniele Cammelli, Maria C. Maggio, Renzo Marcolongo, Francesco La Torre, Claudia Fabiani, Serena Colafrancesco, Francesca Ricci, Paola Galozzi, Ombretta Viapiana, Elena Verrecchia, Manuela Pardeo, Lucia Cerrito, Elena Cavallaro, Alma N. Olivieri, Giuseppe Paolazzi, Gianfranco Vitiello, Armin Maier, Elena Silvestri, Chiara Stagnaro, Guido Valesini, Marta Mosca, Salvatore de Vita, Angela Tincani, Giovanni Lapadula, Bruno Frediani, Fabrizio De Benedetti, Florenzo Iannone, Leonardo Punzi, Carlo Salvarani, Mauro Galeazzi, Donato Rigante, Luca Cantarini

**Affiliations:** ^1^Department of Medical Sciences, Surgery and Neurosciences, Research Center of Systemic Autoinflammatory Diseases and Behçet's Disease Clinic, University of SienaSiena, Italy; ^2^Division of Rheumatology, Department of Pediatric Medicine, IRCCS, Bambino Gesù Children's HospitalRome, Italy; ^3^Rheumatology Unit, Department of Medicine, University of PaduaPadua, Italy; ^4^Rheumatology Unit, Interdisciplinary Department of Medicine, University of BariBari, Italy; ^5^Department of Experimental and Clinical Medicine, University of FlorenceFlorence, Italy; ^6^Pediatric Clinic, University of Brescia and Spedali Civili di BresciaBrescia, Italy; ^7^Periodic Fever Research Center, Institute of Internal Medicine, Università Cattolica Sacro Cuore, Fondazione Policlinico A. GemelliRome, Italy; ^8^Pediatric Rheumatology Unit, AOU MeyerFlorence, Italy; ^9^Department of Internal Medicine and Medical Specialities, Rheumatology Unit, Sapienza University of RomeRome, Italy; ^10^Rheumatology Unit, Department of Clinical and Experimental Medicine, University of PisaPisa, Italy; ^11^Department of Medical and Biological Sciences, Rheumatology Clinic, University of UdineUdine, Italy; ^12^Rheumatology and Clinical Immunology, Spedali Civili, and Department of Clinical and Experimental Sciences, University of BresciaBrescia, Italy; ^13^Department of Pediatrics, Azienda G. Martino, University of MessinaMessina, Italy; ^14^Rheumatology Unit, Department of Internal Medicine, Azienda Ospedaliera ASMN, Istituto di Ricovero e Cura a Carattere ScientificoReggio Emilia, Italy; ^15^Department of Pediatrics, University Federico II of NaplesNaples, Italy; ^16^Rheumatology Section, Immunoallergology Unit, AOU CareggiFlorence, Italy; ^17^Universitary Department “Pro.S.A.M.I.”, University of PalermoPalermo, Italy; ^18^Clinical Immunology, Department of Medicine, University of PaduaPadua, Italy; ^19^Department of Pediatrics, A. Perrino HospitalBrindisi, Italy; ^20^Department of Ophthalmology, Humanitas Research HospitalMilan, Italy; ^21^Rheumatology Section, Department of Medicine, University of VeronaVerona, Italy; ^22^Dipartimento della Donna, del Bambino e di Chirurgia Generale e Specialistica, Seconda Università degli Studi of NaplesNaples, Italy; ^23^Department of Rheumatology, Santa Chiara HospitalTrento, Italy; ^24^Experimental and Clinical Medicine Department, University of FlorenceFlorence, Italy; ^25^Struttura Semplice di Reumatologia, Ospedale di BolzanoBolzano, Italy; ^26^Periodic Fever Research Center, Institute of Pediatrics, Università Cattolica Sacro Cuore, Fondazione Policlinico A. GemelliRome, Italy

**Keywords:** autoinflammatory disorders, treatment, interleukin (IL)-1, anakinra, canakinumab

## Abstract

**Background:** Interleukin (IL)-1 inhibitors have been suggested as possible therapeutic options in a large number of old and new clinical entities characterized by an IL-1 driven pathogenesis.

**Objectives:** To perform a nationwide snapshot of the on-label and off-label use of anakinra (ANA) and canakinumab (CAN) for different conditions both in children and adults.

**Methods:** We retrospectively collected demographic, clinical, and therapeutic data from both adult and pediatric patients treated with IL-1 inhibitors from January 2008 to July 2016.

**Results:** Five hundred and twenty-six treatment courses given to 475 patients (195 males, 280 females; 111 children and 364 adults) were evaluated. ANA was administered in 421 (80.04%) courses, CAN in 105 (19.96%). Sixty-two (32.1%) patients had been treated with both agents. IL-1 inhibitors were employed in 38 different indications (37 with ANA, 16 with CAN). Off-label use was more frequent for ANA than CAN (p < 0.0001). ANA was employed as first-line biologic approach in 323 (76.7%) cases, while CAN in 37 cases (35.2%). IL-1 inhibitors were associated with corticosteroids in 285 (54.18%) courses and disease modifying anti-rheumatic drugs (DMARDs) in 156 (29.65%). ANA dosage ranged from 30 to 200 mg/day (or 1.0–2.0 mg/kg/day) among adults and 2–4 mg/kg/day among children; regarding CAN, the most frequently used posologies were 150mg every 8 weeks, 150mg every 4 weeks and 150mg every 6 weeks. The frequency of failure was higher among patients treated with ANA at a dosage of 100 mg/day than those treated with 2 mg/kg/day (p = 0.03). Seventy-six patients (14.4%) reported an adverse event (AE) and 10 (1.9%) a severe AE. AEs occurred more frequently after the age of 65 compared to both children and patients aged between 16 and 65 (p = 0.003 and p = 0.03, respectively).

**Conclusions:** IL-1 inhibitors are mostly used off-label, especially ANA, during adulthood. The high frequency of good clinical responses suggests that IL-1 inhibitors are used with awareness of pathogenetic mechanisms; adult healthcare physicians generally employ standard dosages, while pediatricians are more prone in using a weight-based posology. Dose adjustments and switching between different agents showed to be effective treatment strategies. Our data confirm the good safety profile of IL-1 inhibitors.

## Introduction

Inhibition of interleukin (IL)-1 was initially adopted for the treatment of rheumatoid arthritis (RA). To date, the receptor antagonist anakinra (ANA), the selective inhibitor of IL-1β canakinumab (CAN), the soluble decoy IL-1-receptor rilonacept, and the human-engineered monoclonal anti-IL-1β gevokizumab represent the four IL-1 inhibitors (IL-1-INH) available (Finch and Sleeman, 2015). However, only the first two agents, ANA and CAN, have been approved for clinical use in Europe.

Since its introduction in 2001 for RA, a number of other inflammatory pathologies have found to benefit from IL-1-INH, in particular monogenic autoinflammatory diseases (AIDs), including familial Mediterranean fever (FMF; Ben-Zvi and Livneh, 2014; Gül et al., 2015), tumor necrosis factor receptor-associated periodic syndrome (TRAPS; Brizi et al., 2012; La Torre et al., 2015; Lopalco et al., 2015b), mevalonate kinase deficiency/hyper-IgD syndrome (van der Hilst and Frenkel, 2010), and cryopyrin-associated periodic syndrome (CAPS; Cantarini et al., 2011; Caorsi et al., 2013; Scarpioni et al., 2015). However, a wide range of polygenic and multifactorial autoinflammatory conditions characterized by at least a partial deregulation of IL-1 have recently been described as responsive to IL-1-INH (Cantarini et al., 2012a,b, 2015c; So et al., 2013; Cavalli and Dinarello, 2015; Lopalco et al., 2015a). Among others, adult-onset Still's disease (AOSD; Naumann et al., 2010; Nordström et al., 2012), systemic juvenile idiopathic arthritis (SOJA; Hedrich et al., 2012; Ruperto et al., 2012a,b), Behçet's disease (BD; Cantarini et al., 2012a,b, 2015b; Vitale et al., 2014; Emmi et al., 2016) and crystal-induced arthritis (So et al., 2007; Schlesinger et al., 2012) are prime examples of multifactorial AIDs showing a good response to IL-1-INH. As a whole, an increasing number of disorders have proven to be characterized by molecular modifications resembling those found in monogenic AIDs. Therefore, the good clinical response shown by monogenic AIDs to IL-1-INH induced clinicians to try the path of IL-1 inhibition in an increasing number of disorders previously labeled as possible polygenic AIDs on the basis of laboratory findings.

Nowadays, in Italy ANA is indicated for the treatment of RA, in association with methotrexate, and CAPS, while CAN is indicated for CAPS, SOJA, and gout. Consequently, the use of IL-1-INH is often done with off-label modality, therefore without the possibility of real and effective monitoring strategies on long-term effectiveness and safety. For this reason, we conducted a multicenter observational study to perform a nationwide evaluation about the use of IL-1-INH for different conditions with both on-label and off-label modalities in order to provide a description of IL-1-INH use in real life and deduce practical implications as reference points for physicians requiring to use IL-1-targeted inhibition.

## Patients and methods

We retrospectively collected demographic, clinical, and therapeutic data from both adult and pediatric patients treated with IL-1-INH from January 2008 to July 2016 in 23 Italian reference Centers for pediatric and adult patients. Collected data included patients' age, gender, disease, disease duration, age at disease onset, response to IL-1-INH, previous and concomitant treatments, dosages employed, and modifications of dosages or frequency of administration, duration of treatment, and causes for discontinuation, including adverse events (AEs) and severe AEs (SAEs).

The primary aims of our study were: (i) to identify the frequency of ANA and CAN prescription as approach in on-label and off-label use; (ii) to describe the percentage of patients needing to switch from ANA to CAN and vice versa focusing the reasons for switching due to AEs, loss of efficacy, primary inefficacy; (iii) to identify the clinical outcome after switching from one to another agent; (iv) to investigate whether dosage adjustments can contribute to the achievement of a secondary response to treatment; (v) to describe the IL-1-INH safety profile and identify any correlation between the age of patients and the occurrence of AEs based to their severity; (vi) to highlight reasons for discontinuation.

The secondary aims were to describe: (i) different dosages employed for ANA and CAN, distinguishing between pediatric and adult subjects; (ii) the number of biologic agents administered before starting IL-1 inhibition; (iii) previous and concomitant use of corticosteroids and disease modifying anti-rheumatic drugs (DMARDs) differentiating by age; (iv) different therapeutic indications differentiating by age.

Describing the frequency of complete response, partial response, and failure to IL-1-INH represented an ancillary end-point.

Response to IL-1-INH was graded as complete, partial, or failing. The evaluation of response was not standardized, however the normalization of inflammatory markers (erythrocyte sedimentation rate, ESR, <15 mm/h; C-reactive protein, CRP, level <0.5 mg/dl) and the disappearance of all previously identified signs and symptoms were considered as criteria for a complete response. Partial response was retained for patients with clinical improvement, but not fulfilling the criteria for complete response. Finally, a treatment was labeled as failing when neither clinical nor laboratory improvements were observed.

For comparisons between adults and children, patients were classified as pediatric when aged <16 years.

The study was approved and reviewed by the local Ethical Committee (AOUS, Azienda Ospedaliera Universitaria Senese) and was conducted according to the declaration of Helsinki.

### Statistical analysis

Descriptive statistics was evaluated for sample size, mean, and standard deviation for quantitative variables. For quantitative data, pair wise comparisons were performed by means of unpaired *t*-test for parametric data and Mann-Whitney *U*-test for non-parametric data after assessing data normality by using Anderson–Darling test. For qualitative data comparisons were performed by means of Chi-square or Fisher's exact test when required. Significance was defined as *p* < 0.05.

## Results

We evaluated 526 treatment courses administered to 475 patients (195 males; 280 females) who underwent IL-1-INH between January 2008 and July 2016. The mean ± *SD* age of patients was of 36.36 ± 22.18 years, the mean ± *SD* age at symptom onset and at diagnosis were of 24.47 ± 19.99 and 29.31 ± 20.18 years, respectively. Patients aged <16 years were 111 (23.4%), corresponding to 135 (25.7%) treatment courses, 93 of which with ANA (68.9%) and 42 (31.1%) with CAN. The mean ± *SD* age of pediatric patients was 10.2 ± 3.8 years (range 1.75–16.0 years). Patients aged more than 16 years were 364. The mean ± *SD* age of adults was 44.8 ± 18.7 (range 16.75–89.0 years). Table 1 shows demographic and clinical data of all patients enrolled, also distinguishing by different therapeutic indications.

**Table 1:** **Demographic and clinical data of all patients enrolled in the study**.

	**N° patients**	***M* (%)**	**Pediatric patients (%)**	**Mean age ± *SD* (years)**	**Age at onset mean ± *SD* (years)**	**Age at diagnosis mean ± *SD* (years)**	**Diagnostic delay mean ± *SD* (years)**
All	475	195 (41.05%)	111 (23.36%)	36.46 ± 22.13	24.44 ± 20.05	29.36 ± 20.19	4.89 ± 9.60
AOSD	78	27 (34.61%)	0 (0%)	47.33 ± 16.03	39.95 ± 16.15	41.82 ± 15.93	1.87 ± 5.38
SOJA	72	34 (47.22%)	53 (73.61%)	11.98 ± 5.29	6.71 ± 4.62	7.07 ± 4.62	0.21 ± 0.43
BD	46	17 (36.95%)	1 (2.17%)	39.54 ± 13.32	27.41 ± 12.60	31.24 ± 12.41	3.83 ± 6.03
RA	42	9 (21.42%)	0 (0%)	67.55 ± 12.51	44.90 ± 12.05	45.95 ± 11.83	1.23 ± 2.21
FMF	34	11 (32.35%)	4 (11.76%)	41.79 ± 19.77	18.96 ± 15.54	36.07 ± 19.31	17.11 ± 15.40
USAID	32	13 (40.62%)	19 (59.37%)	19.42 ± 15.12	8.17 ± 12.87	15.00 ± 14.86	6.83 ± 9.87
CAPS	30	19 (63.33%)	9 (30%)	33.86 ± 21.44	19.22 ± 20.56	28.38 ± 21.77	8.84 ± 13.20
TRAPS	29	12 (41.37%)	3 (10.34%)	39.18 ± 17.25	20.05 ± 15.82	33.68 ± 17.01	13.56 ± 15.23
IRAP	23	13 (56.52%)	4 (17.39%)	40.17 ± 21.43	34.65 ± 22.25	36.17 ± 21.96	1.52 ± 3.98
CRMO	11	3 (27.27%)	6 (54.54%)	13.91 ± 5.01	9.48 ± 5.03	10.59 ± 5.17	1.11 ± 1.75
OTHERS	78	37 (47.43%)	12 (15.38%)	36.46 ± 22.13	24.44 ± 20.05	29.36 ± 20.19	4.73 ± 9.60

*AOSD, Adult Onset Still's Disease; BD, Behçet's Disease; CAPS, Cryopyrin-Associated Periodic Syndrome; CRMO, Chronic Recurrent Multifocal Osteomyelitis; FMF, Familial Mediterranean Fever; IRAP, Idiopathic Recurrent Acute Pericarditis; RA, Rheumatoid Arthritis; SOJA, Systemic Onset Juvenile Idiopathic Arthritis; TRAPS, Tumor Necrosis Factor Receptor-Associated Periodic Syndrome; USAID, Undifferentiated Systemic AutoInflammatory Disease; M, Male; SD, Standard Deviation*.

**Figure 1:**
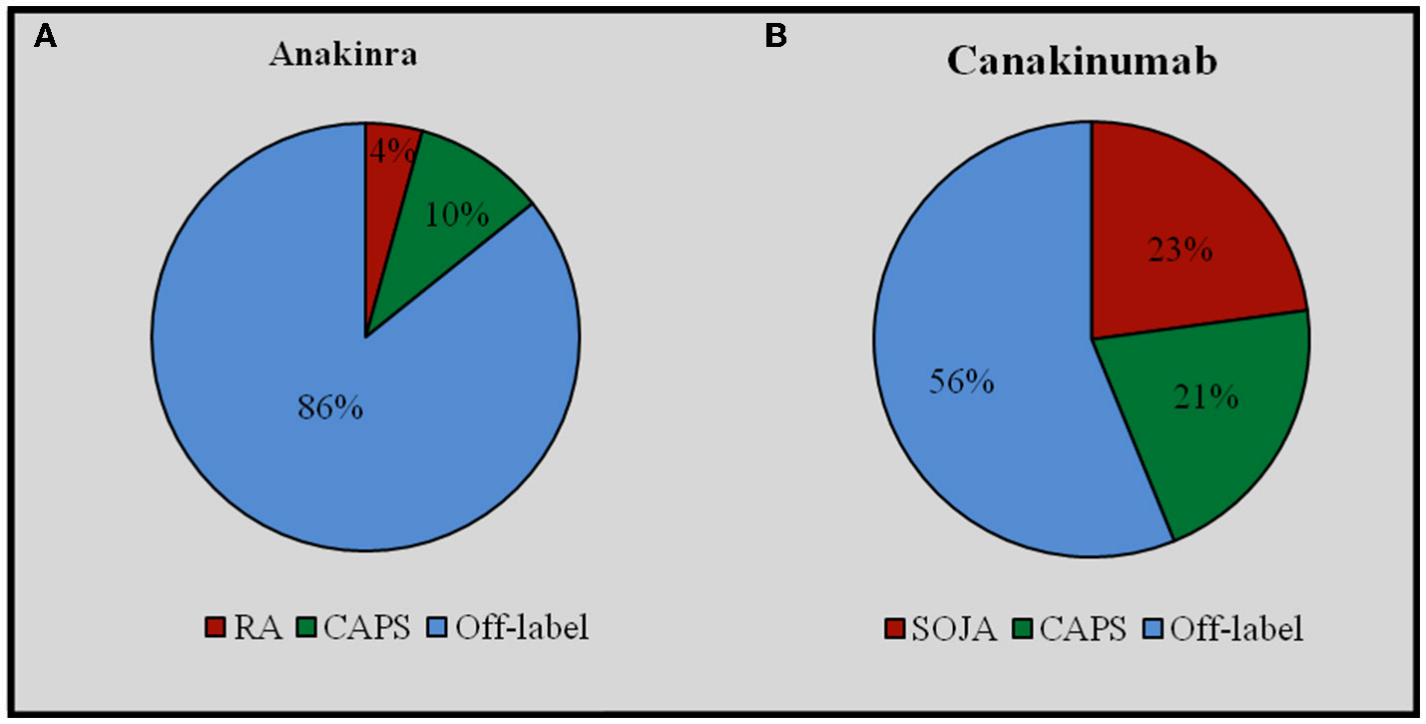
**On-label and off-label use of Anakinra (A) and Canakinumab (B)**. CAPS, Cryopyrin-Associated Periodic Syndrome; RA, rheumatoid arthritis; SOJA, Systemic Onset Juvenile Idiopathic Arthritis.

Overall, ANA was administered in 421 (80.04%) courses and CAN in 105 (19.96%) courses. Sixty-two (32.1%) patients had been treated with both IL-1-INH. ANA was prescribed on-label in 60 (14.3%) cases, while CAN was prescribed on-label in 46 (43.8%) cases. Off-label prescribing was significantly more frequent for ANA than CAN (*p* < 0.0001). Figure 1 graphically describes differences in the on-label use of ANA and CAN.

**Figure 2:**
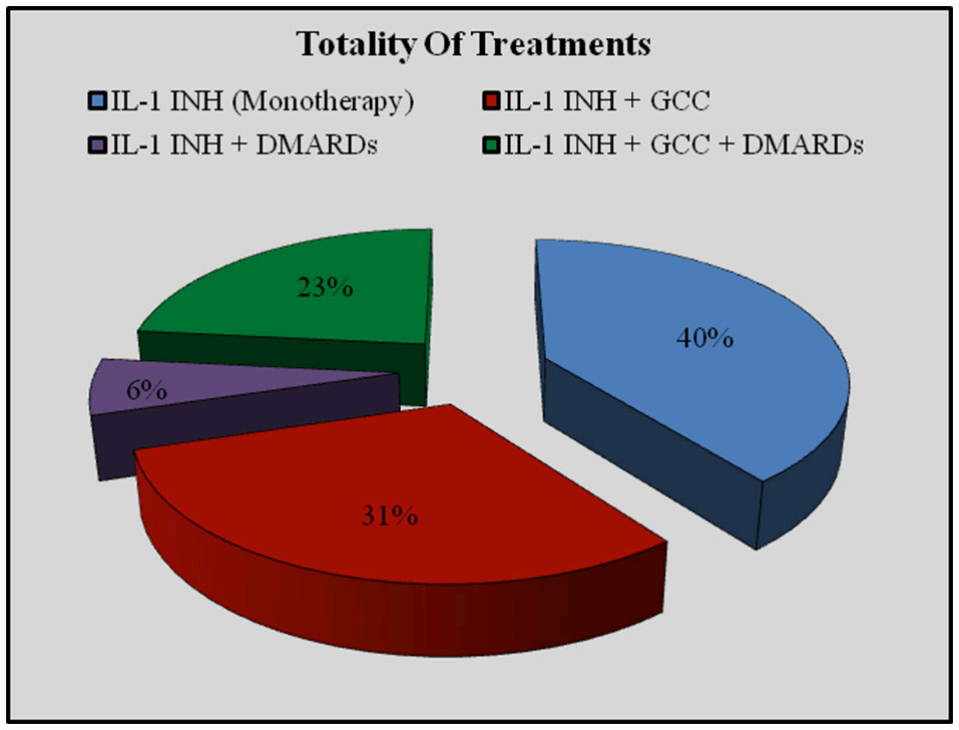
**Use of concomitant therapies during IL-1 inhibition on the whole of treatment courses**. DMARDs, disease modifying antirheumatic drugs; GCC, glucocorticosteroids; IL-1-INH, IL-1 inhibitors.

**Figure 3:**
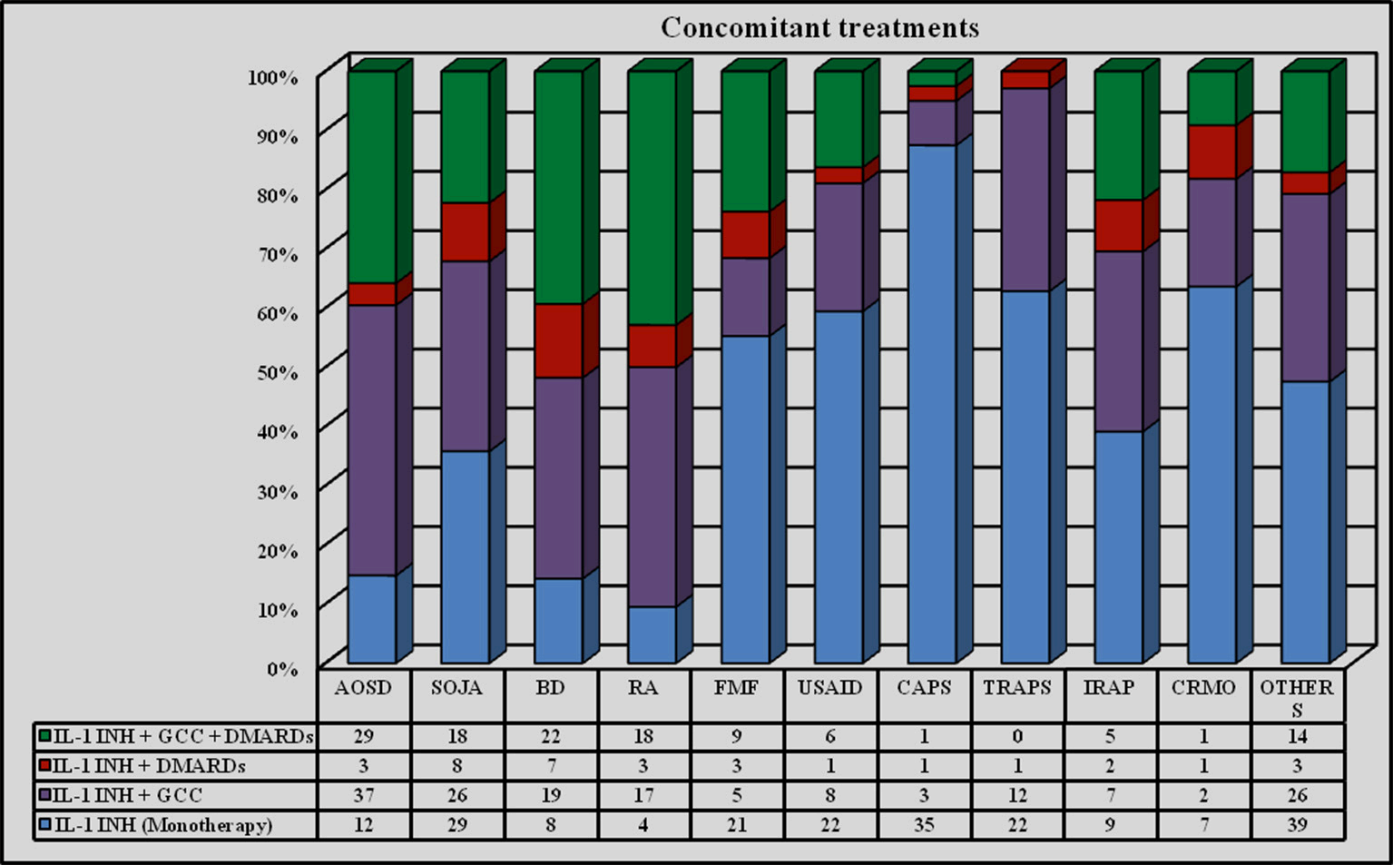
**Use of concomitant therapies during IL-1 inhibition distinguishing by different indications**. AOSD, Adult Onset Still's Disease; BD, Behçet's Disease; CAPS, Cryopyrin-Associated Periodic Syndrome; CRMO, Chronic Recurrent Multifocal Osteomyelitis; FMF, Familial Mediterranean Fever; IRAP, Idiopathic Recurrent Acute Pericarditis; RA, Rheumatoid Arthritis; SOJA, Systemic Onset Juvenile Idiopathic Arthritis; TRAPS, Tumor Necrosis Factor Receptor-Associated Periodic Syndrome; USAID, Undifferentiated Systemic AutoInflammatory Disease.

IL-1-INH were associated with corticosteroids in 285 (54.18%) courses (276 patients, 52.5%) and DMARDs in 156 (29.65%) courses (151 patients, 31.8%). Distinguishing by age, IL-1-INH *plus* corticosteroids were administered to 46 (41.4%) pediatric patients, corresponding to 62 (45.2%) pediatric treatment courses (46 with ANA and 16 with CAN), and 214 (51.6%) adult patients, corresponding to 223 (57.03%) treatment courses (198 with ANA and 25 with CAN). Concomitant corticosteroids were significantly more frequently used among adults than children (*p* = 0.002). DMARDs *plus* IL-1-INH were administered to 27 pediatric patients, corresponding to 32 (23.7%) pediatric treatment courses (23 with ANA and 9 with CAN), and 124 (31.7%) adults, corresponding to 124 (23.6%) treatment courses (115 with ANA and 9 with CAN). Concomitant DMARDs were more frequently administered in adults, without reaching statistical significance (*p* = 0.06). Figure 2 *plus* Figure 3 and Table 2 better specify concomitant therapies during IL-1 inhibition.

**Table 2:** **Previous and concomitant treatments administered to all patients**.

	**Previous treatments**			**Concomitant treatments**	
	**Corticosteroids (%)**	**DMARDs (%)**	**Biologics (%)**	**Corticosteroids (%)**	**DMARDs (%)**
ALL	427/526 (81.17%)	310/526 (58.93%)	165/526 (31.36%)	285/526 (54.18%)	156/526 (29.65%)
AOSD	77/81 (95.06%)	60/81 (74.07%)	17/81 (20.98%)	66/81 (81.48%)	32/81 (39.50%)
SOJA	58/81 (71.60%)	38/81 (46.91%)	22/81 (27.16%)	44/81 (54.32%)	26/81 (32.09%)
BD	44/56 (78.57%)	43/56 (76.78%)	33/56 (58.92%)	41/56 (73.21%)	29/56 (51.78%)
RA	42/42 (100%)	42/42 (100%)	22/42 (52.38%)	35/42 (83.33%)	21/42 (50%)
FMF	30/38 (78.37%)	23/38 (59.45%)	13/38 (35.13%)	14/38 (36.84%)	12/38 (31.57%)
USAID	27/37 (72.97%)	23/37 (62.16%)	10/37 (27.02%)	14/37 (37.83%)	7/37 (18.91%)
CAPS	26/40 (65%)	16/40 (72.97%)	14/40 (35%)	4/40 (10%)	2/40 (5%)
TRAPS	27/35 (77.14%)	3/35 (8.57%)	9/35 (25.71%)	12/35 (34.28%)	1/35 (2.85%)
IRAP	21/23 (91.30%)	11/23 (47.82%)	0/23 (0.00%)	12/23 (52.17%)	7/23 (30.42%)
CRMO	7/11 (63.63%)	3/11 (27.27%)	1/11 (9.09%)	3/11 (27.27%)	2/11 (18.18%)
OTHERS	69/82 (81.36%)	48/82 (58.93%)	24/82 (31.36%)	40/82 (48.78%)	17/82 (20.73%)

*AOSD, Adult Onset Still's Disease; BD, Behçet's Disease; CAPS, Cryopyrin-Associated Periodic Syndrome; CRMO, Chronic Recurrent Multifocal Osteomyelitis; FMF, Familial Mediterranean Fever; IRAP, Idiopathic Recurrent Acute Pericarditis; RA, Rheumatoid Arthritis; SOJA, Systemic Onset Juvenile Idiopathic Arthritis; TRAPS, Tumor Necrosis Factor Receptor-Associated Periodic Syndrome; USAID, Undifferentiated Systemic AutoInflammatory Disease; DMARDs, Disease Modifying Antirheumatic Drugs*.

**Figure 4:**
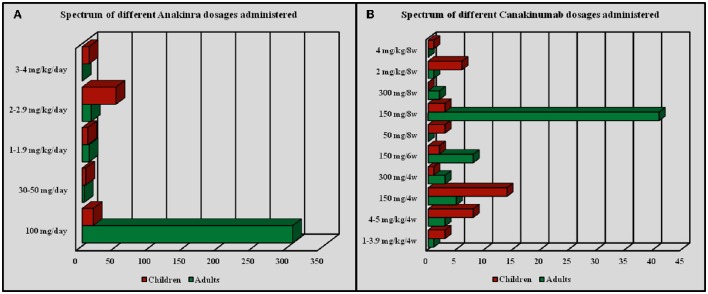
**Frequency of administration for different dosages employed with Anakinra (A) and Canakinumab (B)**.

ANA dosage ranged from 30 mg/day subcutaneously to 200 mg/day (or 1.0–2.0 mg/kg/day) among adults and 2–4 mg/kg/day among pediatric patients; however, 18 out of 28 (64.3%) patients aged from 13 to 16 years were administered the standard dose of 100 mg/day, as more frequently described for adults. One 15-year-old female patient diagnosed with undifferentiated connective tissue disease and suffering from macrophage activation syndrome was treated with ANA at a dosage of 200 mg/day for 8 months. Specifically, the most frequently employed ANA dosages were as follows: 100 mg/day in 322 out of 421 cases (76.5%) as standard posology, 2 mg/kg/day in 64 patients (15.2%), 1–2 mg/kg/day in 18 (4.3%) subjects based on the patient's body weight. Regarding CAN, the most frequently employed posologies were as follows: 150 mg every 8 weeks in 41 out of 105 (39.04%) cases; 150 mg every 4 weeks in 19 patients (18.1%); 150 mg every 6 weeks in 10 patients (9.5%); 4–5 mg/kg every 4 weeks in 11 (10.5%) patients; other dosages (1–4 mg/kg every 4 weeks, 2 mg/kg every 8 weeks, 300 mg every 4 weeks) were employed in 4 (3.8%) subjects. Finally, a patient with CAPS and a second patient with mevalonate kinase deficiency were treated with CAN at a dosage of 300 mg every 8 weeks and a 2-year-old patient diagnosed with CAPS was treated with CAN at the dosage of 4 mg/kg every 8 weeks. Figure 4 summarizes the frequency of administration of different dosages employed for ANA and CAN.

Regarding response, ANA showed complete effectiveness in 256/421 (60.8%) treatment courses and partial effectiveness in 116 (27.6%) subjects, while ANA led to no response in 49 (11.6%). Distinguishing by age, among pediatric patients ANA was completely effective in 66/93 (71%) cases, partially effective in 23/93 (24.7%) cases, and led to no response in 4 (4.3%) cases. In adults, ANA was completely effective in 190/328 (57.9%) treatment courses and partially effective in 93 (28.4%), while 45 (13.7%) patients proved no response and 2 (0.6%) were lost at follow-up. Regarding CAN, among children a complete response was achieved in 26/42 (61.9%) patients, while a partial response was obtained in 14 (33.3%) subjects; failure was identified in 2 (4.8%) patients. Among adults, CAN led to complete response in 38/63 (60.3%) patients and partial response in 19 (30.2%), while failure was observed in 6 (9.5%) subjects. Table 3 summarizes information about complete or partial response, and treatment failure in adults and children treated with ANA and CAN, distinguished by the pertinent diagnosis.

**Table 3:** **Response to IL-1 inhibition among adult and child patients**.

	**Anakinra**						**Canakinumab**					
	**Children (93)**			**Adults (328)**			**Children (42)**			**Adults (63)**		
	**CR**	**PR**	**F**	**CR**	**PR**	**F**	**CR**	**PR**	**F**	**CR**	**PR**	**F**
ToT	66/93 (70.96%)	23/93 (24.74%)	4/93 (4.3%)	190/328 (57.92%)	93/328 (28.35%)	45/328 (13.71%)	26/42 (61.90%)	14/42 (33.33%)	2/42 (4.76%)	38/63 (60.31%)	19/63 (30.16%)	6/62 (9.53%)
AOSD	0/0 (0%)	0/0 (0%)	0/0 (0%)	61/78 (78.20%)	10/78 (12.82%)	7/78 (8.97%)	0/0 (0%)	0/0 (0%)	0/0 (0%)	2/3 (66.66%)	1/3 (33.33%)	0/3 (0%)
SOJA	38/44 (86.36%)	4/44 (9.09%)	2/44 (4.54%)	12/13 (92.3%)	1/13 (7.4%)	0/13 (0%)	12/20 (60%)	7/20 (35%)	1/20 (5%)	3/4 (75%)	1/4 (25%)	0/4 (0%)
BD	0/1 (0%)	1/1 (100%)	0/1 (0%)	15/40 (37.5%)	19/40 (47.5%)	6/40 (15%)	0/1 (0%)	1/1 (100%)	0/1 (0%)	7/14 (50%)	6/14 (42.85%)	1/14 (7.14%)
RA	0/0 (0%)	0/0 (0%)	0/0 (0%)	12/42 (28.57%)	24/42 (57.14%)	6/42 (14.28%)	0/0 (0%)	0/0 (0%)	0/0 (0%)	0/0 (0%)	0/0 (0%)	0/0 (0%)
FMF	1/3 (33.33%)	2/3 (66.66%)	0/3 (0%)	13/29 (44.82%)	9/29 (31.03%)	7/29 (24.13%)	0/2 (0%)	1/2 (50%)	1/2 (50%)	3/4 (75%)	1/4 (25%)	0/4 (0%)
USAID	13/19 (68.42%)	6/19 (31.57%)	0/19 (0%)	6/12 (50%)	3/12 (25%)	3/12 (25%)	3/4 (75%)	1/4 (25%)	0/4 (0%)	1/2 (50%)	1/2 (50%)	0/2 (0%)
TRAPS	1/1 (100%)	0/1 (0%)	0/1 (0%)	14/20 (70%)	5/20 (25%)	1/20 (5%)	3/3 (100%)	0/3 (0%)	0/3 (0%)	8/11 (72.72%)	3/11 (27.27%)	0/11 (0%)
CAPS	5/6 (83.33%)	1/6 (16.66%)	0/6 (0%)	8/12 (66.66%)	4/12 (33.33%)	0/12 (0%)	5/7 (71.43%)	2/7 (28.57%)	0/7 (0%)	11/15 (73.33%)	1/15 (6.66%)	3/15 (20%)
IRAP	3/4 (75%)	1/4 (25%)	0/4 (0%)	15/19 (78.94%)	3/19 (15.78%)	1/19 (5.26%)	0/0 (0%)	0/0 (0%)	0/0 (0%)	0/0 (0%)	0/0 (0%)	0/0 (0%)
CRMO	1/6 (16.66)	3/6 (50%)	2/6 (33.33%)	3/5 (60%)	1/5 (20%)	1/5 (20%)	0/0 (0%)	0/0 (0%)	0/0 (0%)	0/0 (0%)	0/0 (0%)	0/0 (0%)
OTHERS	4/9 (44.44%)	5/9 (55.55%)	0/9 (0%)	31/58 (53.44%)	14/58 (24.13%)	13/58 (22.41%)	3/5 (60%)	2/5 (40%)	0/5 (0%)	3/10 (30%)	5/10 (50%)	2/10 (20%)

*AOSD, Adult Onset Still's Disease; BD, Behçet's Disease; CAPS, Cryopyrin-Associated Periodic Syndrome; CRMO, Chronic Recurrent Multifocal Osteomyelitis; FMF, Familial Mediterranean Fever; IRAP, Idiopathic Recurrent Acute Pericarditis; RA, Rheumatoid Arthritis; SOJA, Systemic Onset Juvenile Idiopathic Arthritis; ToT, totality of treatments; TRAPS, Tumor Necrosis Factor Receptor-Associated Periodic Syndrome; USAID, Undifferentiated Systemic AutoInflammatory Disease. CR, complete response; PR, partial response; F, failure*.

The frequency of failure was significantly higher among patients treated at a dosage of 100 mg/day than patients treated with 2 mg/kg/day (*p* = 0.03). This finding was not maintained when differentiating by the different treatment indications. Regarding CAN, no statistical differences were identified in complete response, partial response and failure according to the different dosages administered (*p* = 0.43).

The mean ± *SD* duration of treatment was 24.4 ± 27 months for both IL-1-INH, corresponding to 24.34 ± 27.03 months for ANA and 24.52 ± 27.06 months for CAN, as well as 26.6 ± 28.6 months for pediatric patients and 24.39 ± 27.04 months for adults. No significant differences were identified between adults and children regarding treatment duration (*p* = 0.51).

### Therapeutic indications

**Figure 5:**
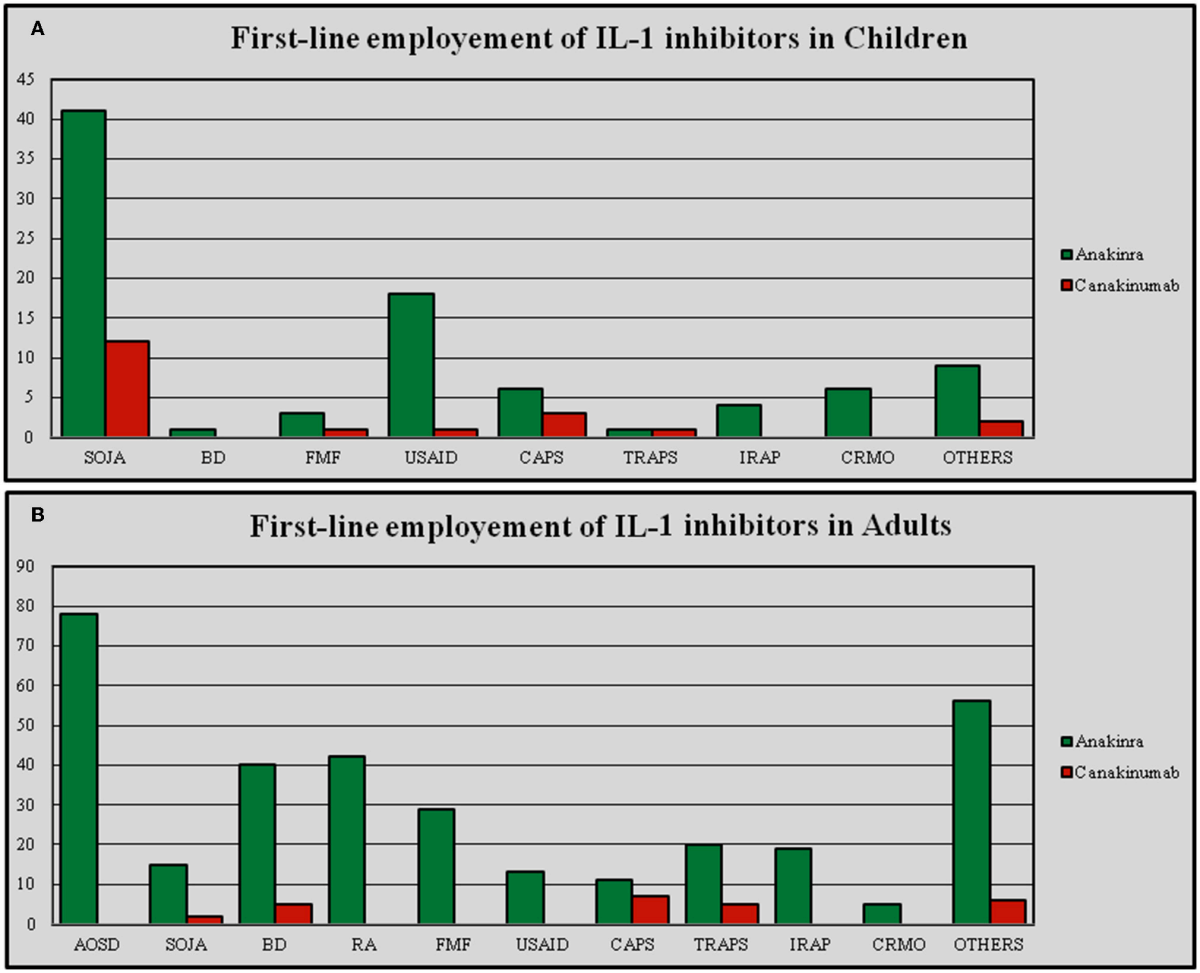
**First-line employment of Anakinra and Canakinumab in different indications differentiating between pediatric (A) and adult patients (B)**. AOSD, Adult Onset Still's Disease; BD, Behçet's Disease; CAPS, Cryopyrin-Associated Periodic Syndrome; CRMO, Chronic Recurrent Multifocal Osteomyelitis; FMF, Familial Mediterranean Fever; IRAP, Idiopathic Recurrent Acute Pericarditis; RA, Rheumatoid Arthritis; SOJA, Systemic Onset Juvenile Idiopathic Arthritis; TRAPS, Tumor Necrosis Factor Receptor-Associated Periodic Syndrome; USAID, Undifferentiated Systemic AutoInflammatory Disease.

As pointed-up in Table 4, IL-1-INH were administered due to 38 different indications, 37 for ANA and 16 for CAN. In pediatric patients IL-1-INH were administered for 16 different indications, 15 for ANA and 10 for CAN; in adults the therapeutic indications were 36, 30 of which for ANA and 15 for CAN. Among patients with complete response the different indications were 25, 23 of which for ANA and 12 for CAN. For patients with partial response the indications were 25, 24 of which for ANA and 12 for CAN. Among patients with failing response the number of indications was 22, 21 of which for ANA and 6 for CAN. The number of indications for IL-1-INH was significantly higher among adults than in pediatric subjects (*p* < 0.0001), but there were no significant differences in the number of indications when ANA and CAN were analyzed separately (*p* = 0.41 and *p* = 0.23, respectively). There were no statistical differences in the number of indications for patients with complete, partial, and failing response neither on the total number of IL-1-INH (*p* = 0.71), nor for ANA (*p* = 0.80) and CAN (*p* = 0.25). When ANA represented the first anti-IL-1 approach, the number of indications stood at 37, while for CAN as the first anti-IL-1 agent the number of indications amounted to 13. Consequently, the number of indications was significantly higher for patients undergoing ANA as first IL-1-INH (*p* < 0.0001); Figure 5 represents the first-line employment of ANA and CAN for the different indications.

**Table 4:** **List of indications for which IL-1 inhibitors were administered**.

**List of indications**	**N° of treatments**
**ANAKINRA (421/526)**	
Adult onset Still's disease	78/421(18.52%)
Ankylosing spondylitis	1/421(0.23%)
Autoinflammatory syndrome induced by adjuvants (ASIA) syndrome	1/421(0.23%)
Behçet's disease	41/421(9.73%)
Blau syndrome	1/421(0.23%)
Chondrocalcinosis	3/421(0.71%)
Chronic recurrent multifocal osteomyelitis	11/421(2.61%)
Cryopyrin-associated periodic syndromes	18/421(4.27%)
Familial Mediterranean fever	32/421(7.6%)
Gout	5/421(1.18%)
Histiocytic panniculitis	1/421(0.23%)
Hyper-IgD syndrome	5/421(1.18%)
Idiopathic recurrent acute pericarditis	23/421(5.46%)
Idiopathic uveitis	2/421(0.47%)
Juvenile idiopathic arthritis	4/421(0.95%)
Mevalonic aciduria	1/421(0.23%)
*NLRP12*-associated familial cold autoinflammatory disease	2/421(0.47%)
Osteoarthritis	1/421(0.23%)
Periodic fever	5/421(1.18%)
Periodic fever, aphthous stomatitis, pharyngitis, cervical adenitis (PFAPA) syndrome	1/421(0.23%)
Polychondritis	1/421(0.23%)
Polyserositis	1/421(0.23%)
Psoriatic arthritis	1/421(0.23%)
PSTPIP1-associated myeloid-related-proteinaemia inflammatory syndrome	1/421(0.23%)
Pyoderma gangrenosum	1/421(0.23%)
Pyogenic arthritis, pyoderma gangrenosum, acne (PAPA) syndrome	2/421(0.47%)
Rheumatoid arthritis	42/421(9.97%)
SAPHO (synovitis, acne, pustulosis, hyperostosis, osteitis) syndrome	3/421(0.71%)
Sarcoidosis	1/421(0.23%)
Schnitzler's syndrome	7/421(1.66%)
Sweet's syndrome	2/421(0.47%)
Systemic-onset juvenile idiopathic arthritis	57/421(13.53%)
Tumor necrosis factor receptor-associated periodic syndrome	21/421(4.98%)
Vasculitic urticaria	1/421(0.23%)
Undifferentiated connective tissue disease	4/421(0.95%)
Undifferentiated spondyloarthritis	1/421(0.23%)
Undifferentiated systemic autoinflammatory disease	31/421(7.36%)
**CANAKINUMAB (105/526)**	
Adult onset Still's disease	3/105(2.85%)
Behçet's disease	15/105(14.28%)
Cryopyrin-associated periodic syndromes	22/105(20.95%)
Epidermolysis bullosa	1/105(0.95%)
Familial Mediterranean fever	6/105(5.71%)
Hyper-IgD syndrome	4/105(3.8%)
Idiopathic uveitis	2/105(1.90%)
Juvenile idiopathic arthritis	2/105(1.90%)
Mevalonic aciduria	1/105(0.95%)
*NLRP12*-associated familial cold autoinflammatory disease	2/105(1.90%)
Periodic fever	1/105(0.95%)
Periodic fever, aphthous stomatitis, pharyngitis, cervical adenitis (PFAPA) syndrome	1/105(0.95%)
Systemic-onset juvenile idiopathic arthritis	24/105(22.85%)
Tumor necrosis factor receptor-associated periodic syndrome	14/105(13.33%)
Vasculitic urticaria	1/105(0.95%)
Undifferentiated systemic auto-inflammatory disease	6/105(5.71%)

### Previous treatments

ANA was employed as first line biologic approach in 323 (76.7%) cases, while CAN was employed as first biologic in 37 cases (35.2%). Consequently, the frequency of ANA administration as first line biologic approach was significantly higher compared to CAN (*p* < 0.0001). ANA and CAN were employed as second biologic line in 52 (12.4%) and 42 (40%) cases, respectively; third line in 28 (6.7%) and 12 (11.4%) patients, respectively; fourth line in 8 cases for ANA (1.9%) and 9 for CAN (8.6%); fifth line in 5 (1.2%) and 4 (3.8%) cases; more than fifth line in 5 (1.2%) and 1 (0.9%) cases. Table 5 displays the clinical outcome for ANA and CAN used for different treatment lines.

**Table 5:** **Percentages of complete response, partial response, and failure to IL-1 inhibition, based on previous biologic therapies**.

**Totality of treatments (526)**	**Type of response**	**0 previous biologics (362/526)**	**1 previous biologic (92/526)**	**2 previous biologics (40/526)**	**3 previous biologics (17/526)**	**4 previous biologics (9/256)**	**≥5 previous biologics (6/256)**
	Complete	243/362 (67.12%)^a^^b^	53/92 (57.60%)^c^^d^	14/40 (35%)^a^^c^	3/17 (17.64%)^b^^d^	4/9 (44.44%)	2/6 (33.33%)
	Partial	83/362 (22.92%)^e^^f^	32/92 (34.78%)	19/40 (47.5%)^e^	10/17 (58.82%)^f^	3/9 (33.33%)	2/6 (33.33%)
	Failure	36/362 (9.94%)	7/92 (7.60%)	7/40 (17.5%)	4/17 (23.52%)	2/9 (22.22%)	2/6 (33.33%)
**Totality of treatments (Anakinra) (421)**	**Type of response**	**0 previous biologics (323/421)**	**1 previous biologic (52/421)**	**2 previous biologics (28/421)**	**3 previous biologics (8/421)**	**4 previous biologics (5/421)**	≥**5 previous biologics (5/421)**
	Complete	219/323 (67.80%)^g^^h^^i^	21/52 (40.38%)^g^	10/28 (35.71%)^h^	2/8 (25%)^i^	2/5 (40%)	2/5 (40%)
	Partial	72/323 (22.29%)^l^^m^^n^	25/52 (48.07%)^l^	13/28 (46.42%)^m^	4/8 (50%)^n^	1/5 (20%)	1/5 (20%)
	Failure	32/323 (9.90%)	6/52 (11.53%)	5/28 (17.85%)	2/8 (25%)	2/5 (40%)°	2/5 (40%)
**Totality of treatments (Canakinumab) (105)**	**Type of response**	**0 previous biologics (37/105)**	**1 previous biologic (42/105)**	**2 previous biologics (12/105)**	**3 previous biologics (9/105)**	**4 previous biologics (4/105)**	≥**5 previous biologics (1/105)**
	Complete	23/37 (62.16%)	33/42 (78.57%)^o^^p^	4/12 (33.33%)^o^	2/9 (22.22%)	2/4 (50%)	0/1 (0%)
	Partial	10/37 (27.02%)^q^	8/42 (19.04%)^r^^s^	6/12 (50%)^r^	6/9 (66.66%)^q^^s^	2/4 (50%)	1/1 (100%)
	Failure	4/37 (10.81%)	1/42 (2.38%)	2/12 (16.66%)	1/9 (11.11%)	0/4 (0%)	0/1 (0%)

a *p = 0.0001*,

b *p < 0.0001*,

c *p = 0.02*,

d *p = 0.0031*,

e *p = 0.001*,

f *p = 0.002*,

g *p = 0.0003*,

h *p = 0.001*,

i *p = 0.0181*,

l *p = 0.0002*,

m *p = 0.009*,

n *p = 0.0850*,

o *p = 0.005*,

p *p = 0.002*,

q *p = 0.04*,

r *p = 0.0573*,

s *p = 0.008*.

**Figure 6:**
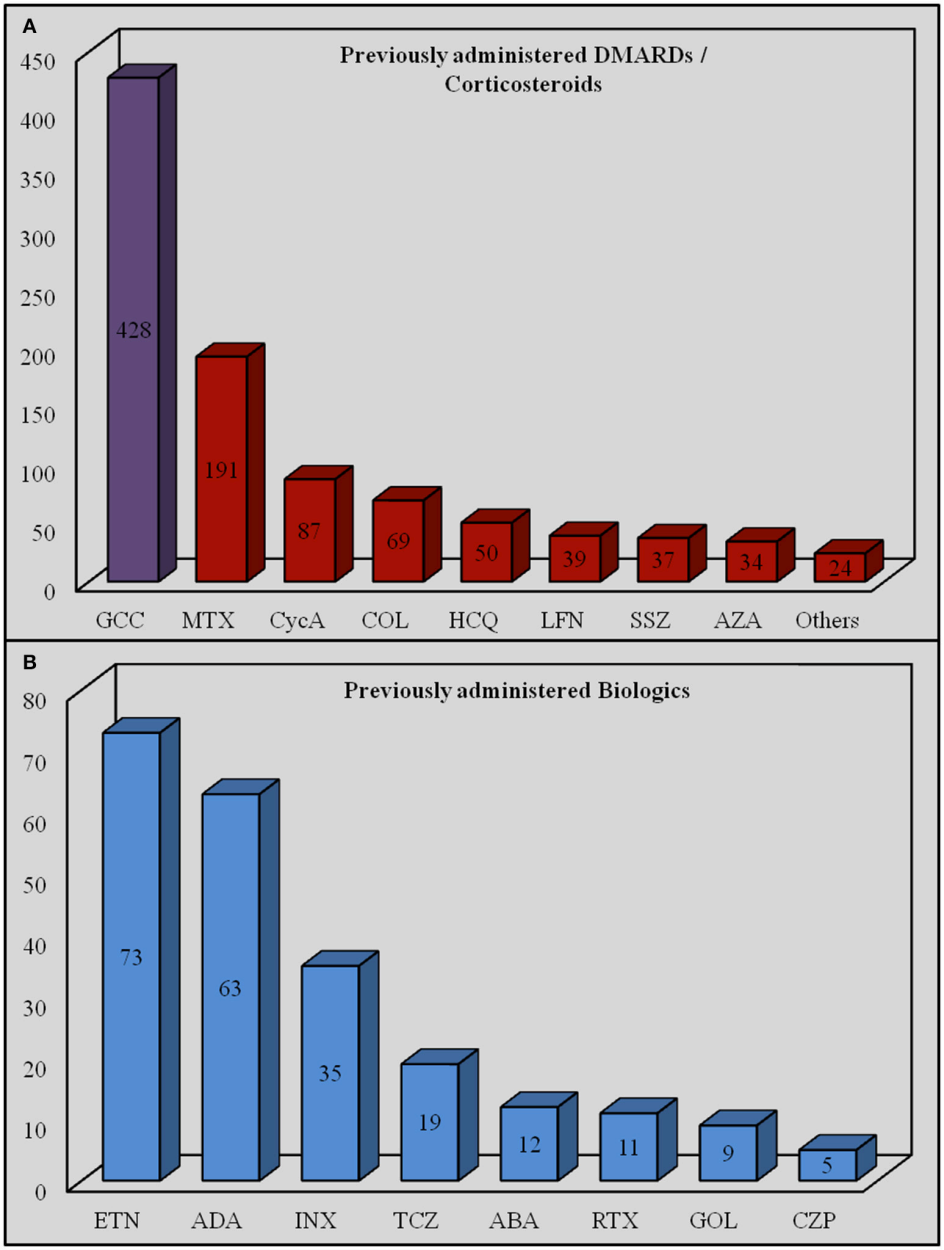
**Amount of specific disease modifying antirheumatic drugs/corticosteroids (A) and biologic agents (B) previously administered**. ABA, abatacept; ADA, adalimumab; AZA, azathioprine; COL, colchicine; CycA, cyclosporine A; CZP, certolizumab pegol; INX, infliximab; ETN, etanercept; GCC, glucocorticoids; GOL, golimumab; HCQ, hydroxychloroquine; LFN, leflunomide; MTX, methotrexate; RTX, rituximab; SSZ, sulfasalazine; TCZ, tocilizumab.

Before starting IL-1-INH, corticosteroids had been already employed in 428 out of 526 (81.4%) cases, 95 (22.2%) of which were pediatric ones; DMARDs had preceded IL-1 inhibition in 309 cases (58.7%), 57 (18.4%) of which were pediatric ones. In addition, 35 pediatric (21.2%) and 130 adult subjects (78.8%) had been previously treated with at least one biologic agent different from IL-1-INH. Table 2 shows details about previous therapeutic approaches, while Figure 6 shows the amount of the specific DMARDs and biologics previously administered.

### Switching from a first to a second anti-Il-1 agent

The number of patients switched from ANA to CAN was significantly higher than patients switched from CAN to ANA (*p* < 0.0001). Specifically, the number of patients firstly treated with ANA and then switched to CAN amounted at 60 (57.1%); conversely, although with a complete response, 2 (0.5%) patients (diagnosed with SOJA and CAPS) needed to be switched from CAN to ANA because of mild leukopenia and loss of efficacy, respectively.

Reasons for switching from ANA to CAN were as follows: loss of efficacy (*n* = 29, 48.3%) after a mean ± *SD* treatment period of 25.97 ± 24.47 months, lack of compliance (*n* = 7, 11.7%), and lack of efficacy (*n* = 6, 10%). However, no data were available about the reason for ANA discontinuation in 18 (30%) patients despite a treatment duration ranging from 3 to 132 months.

Regarding clinical outcome, complete response was achieved in 40 (66.7%) cases switched from ANA to CAN, partial response in 17 (28.3%) and failure in 3 (0.5%). Conversely, both patients switched from CAN to ANA proved to be completely responsive to the second anti-IL-1 agent.

### Concomitant treatments

IL-1-INH were associated with corticosteroids in 285 (54.2%) cases, 244 patients being treated with ANA and 41 with CAN. Concomitant DMARDs were employed in 156 (29.66%) cases, of which 138 (88.46%) were using ANA and 18 (11.54%) CAN. Methotrexate was employed in 67 (42.9%) patients, colchicine in 32 (20.5%), cyclosporine A in 25 (16.03%), hydroxychloroquine in 12 (7.7%), salazopyrine in 7 (4.5%), leflunomide in 6 (3.8%), azathioprine in 6 (3.8%), mycophenolate mofetil in 1 (0.6%) case.

**Figure 7:**
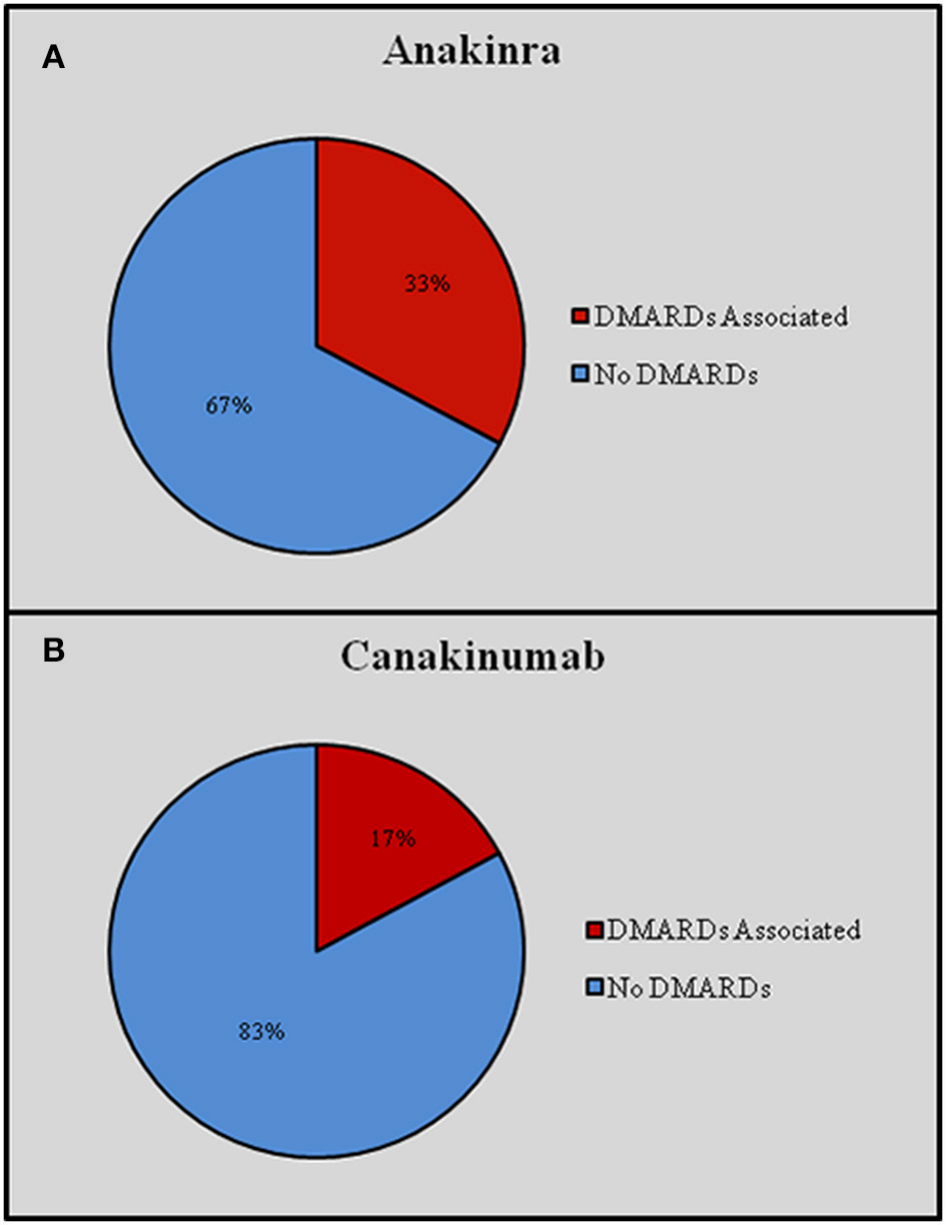
**Frequency of disease modifying antirheumatic drugs (DMARDs) concomitantly administered with Anakinra (A) and Canakinumab (B)**. DMARDs, disease modifying antirheumatic drugs.

In six cases more than one DMARD was associated with IL-1 inhibition: colchicine *plus* hydroxychloroquine, colchicine *plus* methotrexate, methotrexate *plus* leflunomide, methotrexate *plus* cyclosporine A, and salazopyrine *plus* leflunomide each in one case, respectively. Patients requiring two DMARDs were diagnosed with BD (*n* = 3), SOJA (*n* = 1), polyarticular juvenile idiopathic arthritis (*n* = 1), and RA (*n* = 1); these patients were treated with ANA in all cases. Figure 7 graphically shows the frequency of concomitant DMARDs and the distinction according to ANA and CAN use.

### Adverse events

Seventy-six (14.4%) patients reported AEs and 10 (1.9%) SAEs. Specifically, AEs included skin reactions (28 injection site reactions, 5.3%; 29 generalized skin involvement, 5.5%), disorders of hematopoiesis (*n* = 6, 1.15%), infections (*n* = 4, 0.7%), gastrointestinal affections (*n* = 2, 0.3%), flu-like symptoms (*n* = 2, 0.4%), increase of aminotransferases (*n* = 1, 0.2%), thrombophlebitis (*n* = 1, 0.2%), unspecified temporary breathing problems (*n* = 1, 0.2%), unspecified problems (*n* = 2, 0.4%). Seventy-two (94.7%) patients were on ANA and 4 on CAN treatment.

AEs were significantly more frequent among patients with concomitant therapy compared to patients with either concomitant treatments (*p* = 0.01), or previous DMARDs treatment (*p* = 0.04).

Distinguishing among different types of AEs, no significant differences were found on the basis of previous and concomitant therapies; in particular, no difference was found for skin reaction among patients with or without previous DMARDs and/or biologic treatment and patients with or without co-administered DMARDs (*p* = 0.94).

SAEs recorded were as follows: pneumonia in three adult patients with AOSD; trophic ulcers of lower limbs in one patient with AOSD; herpetic keratitis in one patient with RA; and anaphylaxis in four (0.6%). Finally, one patient with BD was diagnosed with pleural mesothelioma after 3 months of ANA treatment. Death occurred in five patients, all treated with ANA. Nine out of 10 patients with SAEs were under ANA treatment. Table 6 summarizes clinical characteristics of patients with SAEs and in which death occurred.

**Table 6:** **General features of patients displaying severe adverse effects (SAEs) and in whom death occurred**.

**N°**	**Gender**	**Age**	**Diagnosis**	**Age at disease onset**	**Number of previous DMARDs**	**Concomitant DMARDs**	**Number of previous biologics**	**Previous GCC**	**Concomitant GCC**	**IL-1 inhibitor**	**Treatment duration (months)**	**SAEs**
**GENERAL FEATURES OF PATIENTS WITH OCCURRENCE OF SEVERE ADVERSE EFFECTS**												
1	M	52	AOSD	49	1	NO	0	YES	NO	Anakinra	17	Pneumonia
2	M	65	AOSD	52	2	YES	1	YES	YES	Anakinra	110	Lower limbs ulcers
3	M	67	AOSD	64	1	YES	1	YES	YES	Anakinra	9	Pneumonia
4	F	15	CAPS	2	1	NO	0	YES	NO	Anakinra	2	Anaphylaxis
5	M	52	FMF	49	1	YES	0	YES	YES	Anakinra	3	Pleural mesothelioma
6	F	76	RA	49	7	NO	2	YES	YES	Anakinra	48	Herpetic keratitis
7	F	64	SAPHO	33	2	YES	4	YES	NO	Anakinra	13	Pneumonia
8	M	15	USAID	0.2	0	NO	0	YES	NO	Anakinra	1	Anaphylaxis
9	F	23	USAID	17	0	NO	0	NO	NO	Anakinra	23	Anaphylaxis
10	M	12.6	SOJA	2	3	NO	0	YES	NO	Canakinumab	80	Anaphylaxis
**N**°	**Gender**	**Age**	**Diagnosis**	**Age at disease onset**	**Number of previous DMARDs**	**Concomitant DMARDs**	**Number of previous biologics**	**Previous GCC**	**Concomitant GCC**	**IL-1 inhibitor**	**Treatment duration (months)**	**Cause of death**
**GENERAL FEATURES OF PATIENTS FOR WHOM DEATH OCCURRED**												
11	F	20	AOSD	17	1	NO	0	YES	YES	Anakinra	n.k.	MAS
12	F	32	AOSD	32	0	NO	0	YES	YES	Anakinra	0.5	Myocarditis
13	F	59	AOSD	49	4	YES	1	YES	YES	Anakinra	120	Dilated cardiomyopathy

*Patients n°3 and n°7 accounted here have to be reported also among patients in whom death occurred. AOSD, Adult Onset Still's Disease; CAPS, Cryopyrin-Associated Periodic Syndrome; FMF, Familial Mediterranean Fever; IRAP, Idiopathic Recurrent Acute Pericarditis; MAS, macrophage activation syndrome; n.k., not known; RA, Rheumatoid Arthritis; SAPHO, Synovitis Acne Pustolosis Hyperostosis and Osteitis syndrome; SOJA, Systemic Onset Juvenile Arthritis; TRAPS, Tumor Necrosis Factor Receptor-Associated Periodic Syndrome; USAID, Undifferentiated Systemic AutoInflammatory Disease; DMARDs, disease-modifying antirheumatic drugs; GCC, glucocorticoids; M, male; F, female*.

**Figure 8:**
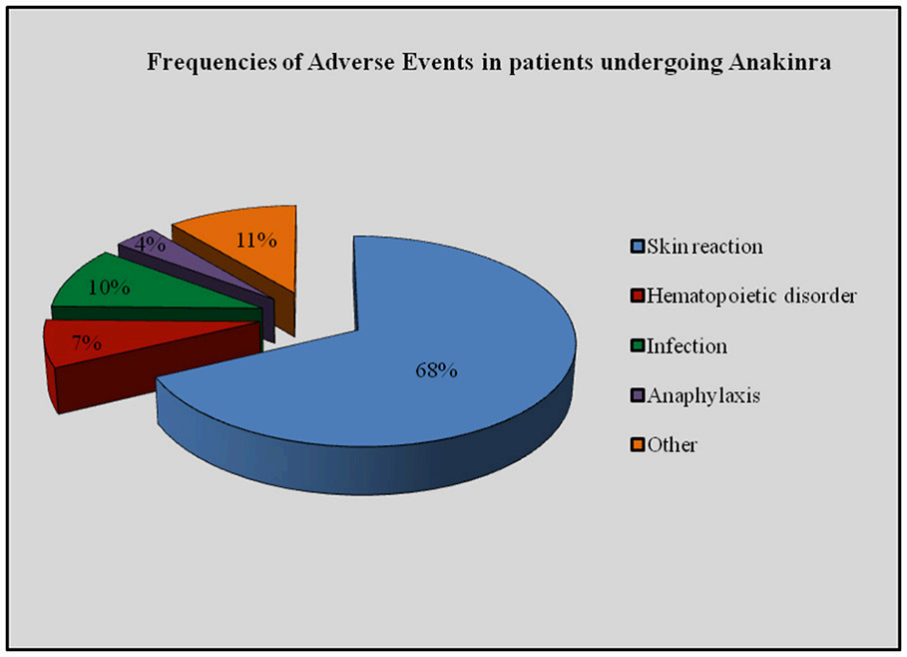
**Frequency of adverse events in patients undergoing Anakinra**.

Figure 8 shows the frequency of AEs in patients undergoing ANA treatment; as regards CAN, skin rash (*n* = 3) and gastrointestinal symptoms (diarrhea, abdominal pain; *n* = 2) represented the most frequently recorded AEs; flu-like symptoms (*n* = 1) and asthenia (*n* = 1) were also reported after CAN administration.

When patients were subdivided into three age groups (<16 years, 16–65 years, and >65 years), AEs proved to be significantly more frequent after the age of 65 compared to both pediatric patients and subjects aged between 16 and 65 (*p* = 0.003 and *p* = 0.03, respectively). Table 7 describes the frequency of different AEs according to the age of patients.

**Table 7:** **Frequency of different adverse events and severe adverse events according to the age of patients**.

**Age (years)**	**N° treatments**	**Total reactions**	**Generalized skin reaction**	**Injection site reaction**	**Hematopoiesis disorders**	**Infection**	**Gastrointestinal involvement**	**Anaphylaxis**	**Other**
**ADVERSE EVENTS AND SEVERE ADVERSE EVENTS (DIVIDED BY AGE)**									
**Total number of AEs: 76 out of 526 treatments**									
All Ages	526	76 (14.44%)	29	28	6	4	2	–	7
0–15.99	130	11 (8.46%)	1	7	–	–	1	–	2
16–64.99	331	50 (15.1%)	22	16	3	4	1	–	4
≥65	65	15 (23.07%)	6	5	3	–	–	–	1
**Total number of SAEs: 10 out of 526 treatments**									
All Ages	526	10 (1.9%)	–	–	–	4	–	4	2
0–15.99	130	3 (2.3%)	–	–	–	–	–	3	–
16–64.99	331	4 (1.2%)	–	–	–	2	–	1	1
≥65	65	3 (4.61%)	–	–	–	2	–	–	1

*AEs, Adverse Events; SAEs, Severe Adverse Events*.

In 61 (71.8%) cases AEs led to treatment discontinuation, in 59 cases treated with ANA and in two cases treated with CAN. Regarding patients remaining under ANA treatment despite AEs, most were characterized by injection site reactions (*n* = 13); others were interested by infections (otitis, *n* = 1; infection of the upper respiratory tract, *n* = 1; bronchitis, *n* = 1; pneumonia, *n* = 1), transient leukopenia (*n* = 1), thrombophlebitis (*n* = 1), widespread skin rash with eosinophilia (*n* = 1). Patients continuing CAN despite AEs had presented localized cutaneous erythema (*n* = 2) and flu-like symptoms (*n* = 1).

### Dose adjustments

Dose adjustments were performed for 117 treatment cycles (22.4%), 88 (75.2%) for ANA and 29 (24.8%) for CAN; no statistical differences were identified in the number of dose adjustments between ANA and CAN (*p* = 0.29).

**Figure 9:**
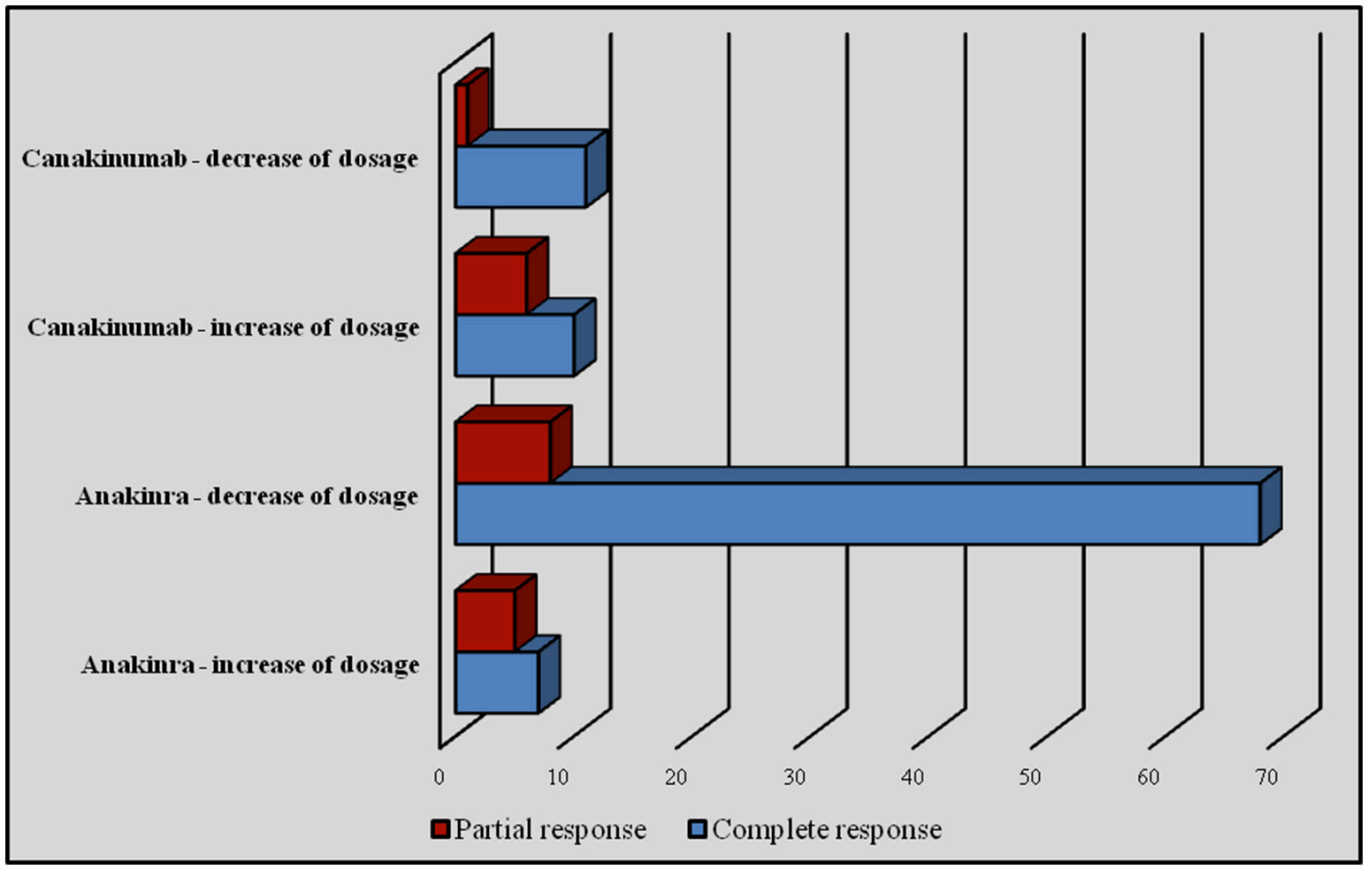
**Number of patients undergoing an increase or decrease of IL-1 INH dosage with related clinical outcome**.

Regarding subjects treated with ANA, an increase of the dose was performed in 12 cases, bringing about a recovery of effectiveness in 7 (58.3%) cases. Conversely, a decrease of the dosage was attempted in 76 patients, leading to maintenance of therapeutic efficacy in 89.4% (*n* = 68) of cases. Among patients treated with CAN, an increase of dosage was performed in 15.2% (*n* = 16) of patients, obtaining a recovery of efficacy in 10 (62.5%). On the contrary, a reduction of dosage was attempted in 12 (11.4%) subjects, leading to maintenance of efficacy in 11 cases out of 12 (91.6%). One patient (0.9%) with TRAPS underwent an increase of the interval between CAN administrations without changing the dosage and without losing efficacy. Figure 9 shows the number of patients undergoing an increase/decrease of IL-1 INH dosage with related clinical outcome.

### Reasons for discontinuation

**Figure 10:**
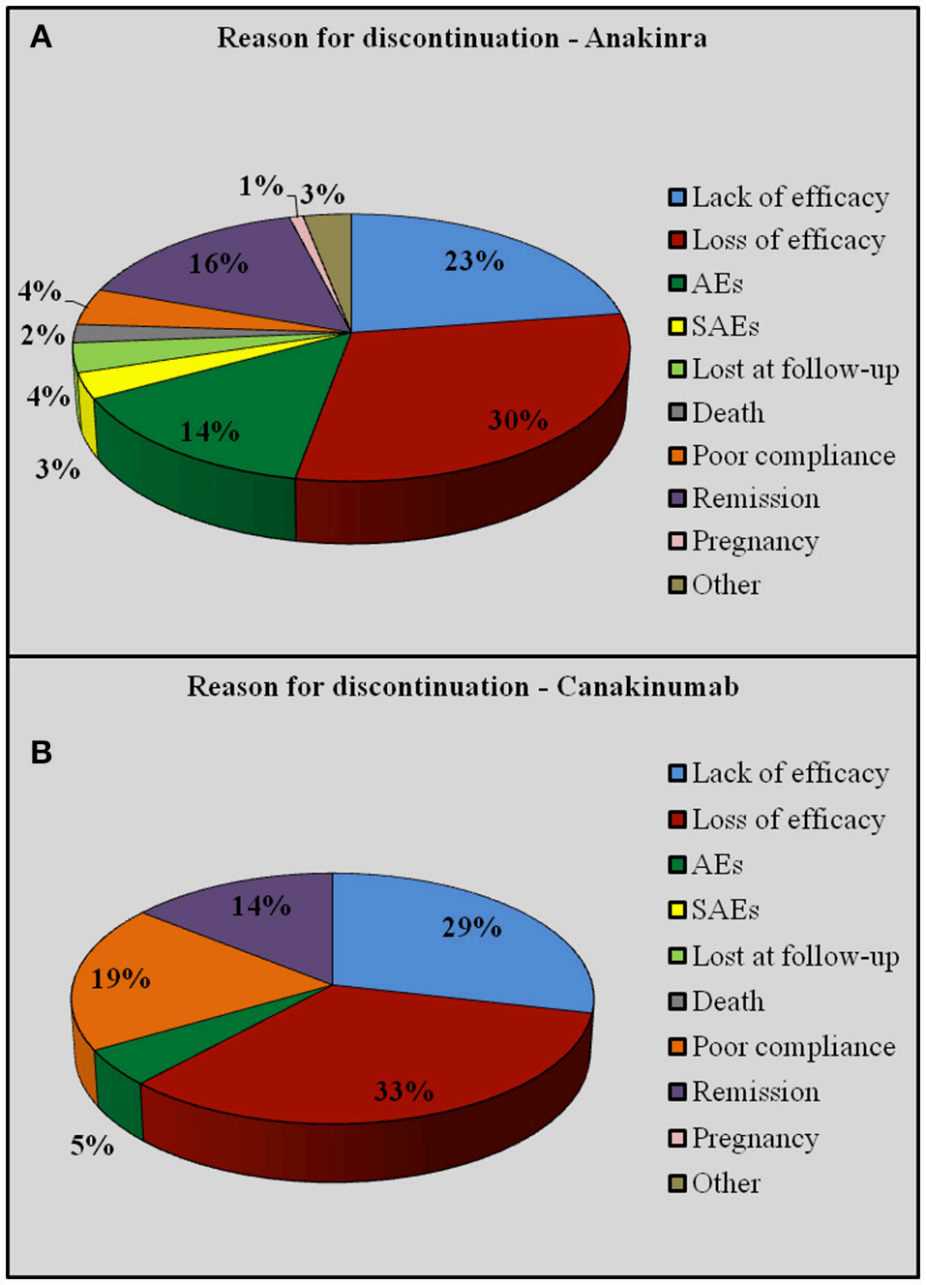
**Reasons for discontinuation distinguishing between Anakinra (A) and Canakinumab (B)**.

Discontinuation of anti-IL-1 regimen was performed in 246 (46.6%) cases, 225 (91.5%) while on ANA and 21 (8.5%) on CAN. The reasons for discontinuation were as follows: loss of efficacy (*n* = 75, 30.4%), lack of efficacy (*n* = 57, 23.2%), disease remission (*n* = 38, 15.4%), occurrence of AEs (*n* = 33, 13.4%) and SAEs (*n* = 7, 2.9%), poor compliance (*n* = 14, 5.6%), death (*n* = 5, 2%), pregnancy (*n* = 2, 0.8%). Eight cases (3.2%) were lost at follow-up. Figure 10 shows the reasons for discontinuation by distinguishing between ANA and CAN. As this figure shows, the percentage of poor compliance is higher in patients treated with CAN, though no statistical significance was reached (*p* = 0.49).

## Discussion

In this study, we aimed at evaluating how IL-1-INH are currently used in a group of Italian rheumatological and pediatric Centers, principally in terms of therapeutic indications, dosages employed, clinical management, and safety issues, thus identifying differences between adults and patients aged <16 years. In particular, by virtue of the large number of patients enrolled, we would provide useful real-life-related data for the physician needing to resort to IL-1 inhibition.

To date, therapeutic indications for ANA include only RA and CAPS, while on-label use of CAN refers to CAPS, SOJA, and gout. Nevertheless, thanks to vivid basic and clinical research efforts, innate immunity has recently proven to have an important role in a wide number of disorders beyond monogenic AIDs (Banerjee and Saxena, 2012; Dickie et al., 2012; Vitale et al., 2012; Caso et al., 2013; Sheedy and Moore, 2013; Van Tassell et al., 2013; Baskar et al., 2016). Sure enough, inflammatory pathways of innate immunity have been found to affect a lot of pathologies previously classified as exclusively belonging to the field of adaptive immune disorders or degenerative diseases (Martinon et al., 2006; Rigante et al., 2011; Kotas et al., 2013; Ruscitti et al., 2015; Thueringer et al., 2015). Consequently, in the last years a large number of old and new clinical entities have been suggested as possible further therapeutic indications for IL-1-INH (Cantarini et al., 2010, 2015b; Caso et al., 2014; Finetti et al., 2014; D'Elia et al., 2015; Imazio et al., 2016). Therefore, whilst now it is absolutely clear that tumor necrosis factor (TNF)-inhibitors represent a therapeutic option of first order compared to IL-1-INH in RA, a resurgence of interest has occurred for these agents to treat a wide number of diseases located somewhere along a continuity of aberrant innate or adaptive immune responses (Larsen et al., 2007; McGonagle et al., 2007; Shin et al., 2009; Tanzi et al., 2011; Abbate et al., 2013; de Koning et al., 2013; Howard et al., 2014; Néel et al., 2014; Van Tassell et al., 2014; Lopalco et al., 2015b, 2016; Vitale et al., 2015; Annicchiarico et al., 2016).

In this context, Rossi-Semerano et al. (2015) have recently published an interesting national cross-sectional observational study on 189 patients from 38 France Centers. As for our results, they also observed that AOSD and SOJA represented the clinical conditions more frequently requiring IL-1-INH; also, ANA was more commonly employed than CAN with off-label modality. Regarding clinical outcome, they found a higher response rate among patients suffering from Schnitzler's syndrome, gout, CAPS, and AOSD. Both ANA and CAN appeared safe as for our patients: most of AEs were classified as minor, while most of the time SAEs were represented by severe infections. However, while Rossi-Semerano et al. (2015) observed a number of patients with liver abnormalities and weight gain, we identified only one case of liver toxicity and did not observe any case of weight increase.

Coming back to our study, in our experience most of treatment courses were due to off-label indications, ranging from different monogenic AIDs to a wide variety of polygenic and multifactorial disorders, as summarized in Table 4. This is evident for both IL-1-INH, but as for Rossi-Semerano et al. (2015), the percentage of off-label use was significantly higher among patients treated with ANA. We think that this is probably related to the high manageability of ANA and to its shorter half-life (Church and McDermott, 2009), but also to the longer experience gained with ANA, and perhaps to cost-related implications. As a whole, the number of different indications for IL-1-INH was significantly higher in adults than in pediatric patients, suggesting that the off-label use of ANA and CAN is more frequently advised by adult health care physicians. However, we did not find statistical differences in the number of indications among patients presenting with complete, partial and failing response, concluding that IL-1-INH are used with awareness of pathogenetic mechanisms also when administered with off-label modality.

In addition to the large number of therapeutic indications, the off-label use of IL-1-INH also manifests with a wide variability of dosages administered both among children and adults. Noteworthy, dosages were more frequently employed based on the body weight among pediatric patients, while adults were more frequently treated with standard dosages. On the contrary, teenage patients (aged <16 years) were frequently treated with a standard dose, as occurring in adults, as a result of the transition from pediatric to adult health care. However, since the body weight affects the pharmacokinetics of drugs (Urien et al., 2013), the two different modalities of IL-1-INH administration may influence the degree of effectiveness. In this regard, we found that the frequency of failure was significantly higher among patients treated with ANA at the dosage of 100 mg/day compared to dosage of 2 mg/kg/day. Conversely, no similar results were identified for CAN, probably due to a lower impact of the body weight related to the much longer half-life (Church and McDermott, 2009), but also to the tiny sample sizes obtained after dividing our CAN population for different dosages.

As expected, we observed that the number of patients switched from ANA to CAN was significantly higher than vice versa, as a likely result of the higher costs of CAN and, here again, of the easy handling of ANA. In fact, the little experience to date available in the off-label context and the relatively scarce supporting literature set physicians advising ANA for safety reasons. Nevertheless, data available on CAN safety (Alten et al., 2011; Ruperto et al., 2012a,b; Imagawa et al., 2013; Howard et al., 2014; Gül et al., 2015), also supported by the present study, make switching from CAN to ANA a reasonable medical choice. In addition, our results demonstrate that patients requiring being moved-on from CAN to ANA showed a complete response after the change of therapy. However, switching between the two IL-1-INH represents a concrete and effective therapeutic opportunity in both directions. Indeed, only 0.5% of patients needing to be switched from ANA to CAN showed a failure after change of therapy, and two-thirds of subjects converted from ANA to CAN even showed a complete response. Consequently, these results confirm previously reported data on the concrete and effective role of switching from a first to a second IL-1-targeted inhibitor (Brizi et al., 2012; Galeotti et al., 2012; Cantarini et al., 2015b; Lopalco et al., 2015b; Emmi et al., 2016).

Similarly, adjusting IL-1-INH dosages by increasing the dose at each administration or decreasing the timing between injections have proved to be successful choices in 66.7% of patients treated with ANA or CAN. These findings suggest that increasing the dosage of IL-1-INH in patients with unsatisfactory results can be a feasible therapeutic strategy. On the other hand, decreasing the dosage in patients with sustained drug-induced quiescence seems to represent another possible way to go when the clinical condition is suitable. Indeed, 92.9% of patients treated experienced maintenance of complete response despite the dose tapering.

Regarding which agent physicians chose as a first option, ANA was employed as first line biologic agent in a greater number of patients, probably due to the higher use of this drug in off-label prescription in reference to the aforementioned explanations. Similarly, the number of DMARDs administered before and during IL-1-INH reflects the frequency with which ANA and CAN were prescribed for complex and multifactorial indications. Actually, the frequency of concomitant and previous DMARDs was higher in adults than pediatric patients and in the population treated with ANA. These findings confirms that IL-1-INH are more likely aimed at treating multifactorial disorders in such cases, whose pathogenesis involves different cell and cytokine pathways and often requires a combination therapy rather than monotherapy. Read backward, these data also suggest that during childhood IL-1-INH (especially CAN) are more frequently used in monogenic AIDs, generally not requiring DMARDs.

As a whole, AEs and SAEs interested one-sixth and 1.7% of patients, thus confirming the good safety profile of IL-1-INH. Interestingly, patients with previous or concomitant use of DMARDs showed a higher frequency of AEs, probably due to the intrinsic safety issues related to the additional employment of DMARDs as well as to the wide number of clinically complex diseases requiring additional immunosuppressive therapies. However, we did not observe any impact of DMARDs on the occurrence of skin reactions. This is an interesting finding, as our results are the opposite of that presented by Rossi-Semerano et al. (2015).

In any case, according to the previous experience with ANA, skin reactions did not lead to treatment discontinuation, confirming that this kind of AEs are usually transient and improves after the first weeks of treatment, with no need for ANA withdrawal (Lequerré et al., 2008).

As we look at SAEs, 40% of them were represented by anaphylactic reactions occurring in three patients treated with ANA and one patient treated with CAN. The other SAEs were severe bacterial infections in three cases, one case of severe herpetic keratitis, and one case of pleural mesothelioma. Death occurred in five cases, two of which as a consequence of infectious SAEs (pneumonia in both cases). However, these infections appeared more likely related to the existing comorbidities and to the globally poor clinical condition than to an actual compromising effect of ANA. In particular, two cases of severe infections had been diagnosed with AOSD and one case with SAPHO syndrome; moreover, AOSD represented the indication requiring IL-1-INH in four out of five cases in whom death occurred. In this regard, the mortality rate in AOSD is reported in up to 10% of patients and overwhelming infections (as also the macrophage activation syndrome and the myocarditis reported in two of our dead patients) represent a major cause of death. Pneumonia itself has been identified as a frequent cause of mortality and an unfavorable prognostic factor in AOSD (Zeng et al., 2009; Kim et al., 2012). Conversely, the dynamics of death of the patient with SAPHO were not completely clear, and we cannot explain in which context pneumonia led to death. Finally, the case of pleural mesothelioma was diagnosed after only 3 months of ANA administration, so we do not ascribe any cancerogenetic effect to IL-1 inhibition.

An intriguing finding of the present study was represented by the significant higher occurrence of AEs in subjects aged more than 65 years than in others, children included. This result conflicts with the evidence reported by Rossi-Semerano et al. (2015), identifying a higher incidence of AEs among children than in adults. Since no notable differences exist between the two studies in terms of percentages of pediatric and adult patients, this difference could be explained by the higher number of indications observed in our population. Consequently, while a careful monitoring should be guaranteed for all patients, elderly subjects could deserve a closer follow-up, especially for poorly investigated indications.

Noteworthy, none of our patients experienced tuberculosis infection or reactivation, corroborating our previous results on the same matter (Cantarini et al., 2015a; Lopalco et al., 2016) and suggesting that use of IL-1-INH is relatively safe compared to other therapeutic tools in those geographical areas where tuberculosis is an endemic issue.

Because of the retrospective design, this study has some limitations: firstly, although data collection was quite exhaustive, some information was lacking due to the inability to collect all clinical data. This was especially true about the reasons for discontinuation, which remained without explanation in 18 cases treated with ANA. Secondly, a limitation of the causality assessment is highly probable in a retrospective study. In addition, the huge number of therapeutic indications made impossible a sharp statistical analysis on the response to IL-1-INH. Indeed, fragmentation of cases among 38 different indications and three possible outcomes (complete response, partial response, and failure) led to small sample sizes and convincing conclusions could not be drawn. As a result, we performed an overall description on the response and placed the issue of clinical response among the ancillary end-points. However, this is the first study showing how and when IL-1-INH are prescribed in Italy, highlighting different therapeutic choices in terms of starting dosages, dose adjustments, and switching from one to another IL-1-INH as well as the assessment of safety profile on a large number of patients.

In conclusion, our data show that treatment with IL-1-INH is mostly used in off-label regimen. Nevertheless, the high amount of complete and partial clinical response obtained suggests that IL-1-INH are administered in clinical conditions mostly characterized by the pathogenetic involvement of IL-1 cytokine network. Accordingly, most of the patients were concomitantly treated with DMARDs and had been previously administered with other biologic agents different from IL-1-INH, especially anti-TNF drugs. The off-label use of IL-1-INH has been more frequent for ANA and for adult patients. The wide spectrum of dosages administered for IL-1-INH is a further interesting information emerging from our data: while adult health care physicians generally employ standard dosages of IL-1-INH, pediatricians are more frequently inclined to use a weight-based posology, which seems to be a more adequate therapeutic strategy because of pharmacokinetic implications about drug-tissue concentrations. Furthermore, switching from a first to a second IL-1-INH and increasing dosages appear to be useful in order to obtain a more successful clinical response. According to our findings, switching from CAN to ANA is a less common therapeutic choice than the reverse. However, patients undergoing this procedure showed complete response, and consequently we think that this therapeutic option should be kept into much greater account. The present study confirms the good safety profile of IL-1-INH in terms of low risk of tuberculosis. In addition, the majority of AEs were mild or moderate and did not require treatment discontinuation. On the other hand, SAEs and deaths reported were mostly connected to the underlying disease or other comorbidities. Finally, our data show that a slightly closer follow-up may be useful in patients over 65 years of age.

## Author contributions

AV, DR, and Luca Cantarini wrote the manuscript. LCa designed the study and finally revised the manuscript. AV and LCa: data analysis. AI, PS, GLo, GE, MC, RMan, RC, RP, RT, SG, GD, MF, RG, AS, MA, DC, MCM, RMar, FL, CF, SC, FR, PG, OV, EV, MP, LCe, EC, AO, GP, GVi, AM, ES, CSt, GVa, MM, SD, AT, GLa, BF, FD, FI, LP, CSa, MG: patients enrollment, follow-up of the patients and data collection.

### Conflict of interest statement

The authors declare that the research was conducted in the absence of any commercial or financial relationships that could be construed as a potential conflict of interest.
